# Substance Abuse and Rural Appalachian Pediatric Trauma in West Virginia

**DOI:** 10.1155/2022/4906812

**Published:** 2022-06-26

**Authors:** Joshua Rawson, Lindsey Thevenin, Isabella Balko, Federico Seifarth, Hal Meltzer, Vipul Dhumak, Amy Bush, Wesley Kimble, Sijin Wen, Pavithra Ellison

**Affiliations:** ^1^School of Medicine, West Virginia University, Morgantown, WV, USA; ^2^West Virginia University, Morgantown, WV, USA; ^3^Department of Surgery, West Virginia University, Morgantown, WV, USA; ^4^Department of Anesthesiology, West Virginia University, Morgantown, WV, USA; ^5^West Virginia Clinical and Translational Research Institute, Morgantown, WV, USA

## Abstract

**Introduction:**

Rural Appalachia is endemic to issues such as substance abuse, poverty, and lack of community support, all of which negatively influence health outcomes. The incidence of pediatric trauma as it relates to substance abuse is of concern in the region, where the rate of positive drug screens in pediatric trauma cases is higher than national average.

**Methods:**

The West Virginia statewide pediatric trauma database was analyzed in a retrospective cohort study for the years 2009-2019. Variables of interest included injury severity (assessed using Abbreviated Injury Scale (AIS)), drug screening results, and various measures of patient outcome.

**Results:**

The sample was divided into 2009-2016 presentations (*n* = 3,356) and 2017-2019 presentations (*n* = 1,182). Incidence of critical (AIS 5) head injuries (*p* = 0.007) and serious (AIS 3) neck injuries (*p* = 0.001) increased as time progressed. Days requiring ventilation increased from 3.1 in 2009–2016 to 6.3 in 2017–2019 (*p* < 0.001). Drug screens were obtained at a rate of 6.9% in 2009–2016 versus 23.3% in 2017–2019 (*p* < 0.001). Benzodiazepine use increased from 0.8% to 1.8% (*p* < 0.001), and opioid use increased from 1% to 4.9% (*p* < 0.001).

**Conclusion:**

The increasing severity of pediatric trauma and substance abuse in Appalachia is of significant concern. The use of respiratory drive-depressing drugs has risen, just as the severity of head and neck traumas has increased. These results emphasize the importance of targeted interventions in the rural pediatric population.

## 1. Introduction

Rural Appalachia, encompassing over 400 counties spanning parts of 13 states, [1], faces many challenges that influence both timely access to medical care and the level of care that is needed by patients. Issues prevalent in the area include substance abuse, poverty, and lack of community support, all of which negatively influence health outcomes and result in increased mortality rates when compared to urban or suburban areas [2]. Further magnifying these problems are the geography of rural Appalachia and its scarcity of healthcare resources, which limit access to care for much of the population. One issue endemic to the area that has garnered the most media attention is substance use, more specifically opioid use [3]. Additionally, the use of stimulants and substances other than opioids that depress the respiratory drive has also created problems for individuals and families [4]. Many children and adolescents have experienced primary and secondary effects of substance abuse including legal troubles, adverse health effects such as physical trauma, mental health disorders, and social issues such as family dissolution [5]. The association between pediatric trauma and substance use has not previously been well-established in literature.

Social issues that are endemic to rural Appalachia, particularly pertaining to substance abuse, may largely be contributing to pediatric trauma incidence and outcome. Nationally, 6.3% of all pediatric trauma cases tested positive for illegal drugs or alcohol levels above the legal limit via urine drug screening [6]. However, a recent study that took place in West Virginia, the only state located entirely in Appalachia, revealed that 7.2% of the pediatric trauma study population tested positive for illegal drugs or alcohol via urine drug screening [2]. The higher prevalence of substance use in the rural areas of Appalachia may impact the incidence and severity of pediatric trauma-related injuries seen in the more rural areas compared to urban and suburban areas.

While it has become more apparent that substance abuse is a problem in the younger population, many children and adolescents suffering from addiction are not being identified and referred for intervention due to undertesting. In a study analyzing a cohort of 157,450 pediatric trauma cases, only 28.9% were screened for substance abuse; 36.7% of patients had a positive result, with cannabinoids, alcohol, and opioids being the substances most commonly detected [7]. While some studies have suggested that the frequency of drug screening in pediatric trauma increases with age of patient [7, 8], low drug screening rates observed nationally in pediatric trauma cases of all ages, including the preadolescent population, have created difficulties that prevent providers from adequately addressing what may often be the primary cause of the trauma.

In this study, we utilized a statewide trauma registry to review pediatric trauma and drug screen data in West Virginia, a state which has served as the epicenter of the opioid epidemic with the highest rate of overdose in the country [9]. Because there is little data available on pediatric substance use and injury severity in trauma cases, all pediatric trauma patients from 2009 to 2019 were analyzed to determine if there was an association between case load and severity of injuries relative to the increasing incidence of substance abuse in the state. We also analyzed the trend of abuse for each substance over that time frame.

## 2. Methods

The West Virginia statewide trauma database was analyzed in a retrospective cohort study design. Pediatric trauma patients, defined as those younger than eighteen, who presented to a trauma center in the state from the years 2009 to 2019 were included in the analysis. Obtained from the database were the following variables: demographic information (patient age and gender), injury mechanism, injury severity (assessed using the Abbreviated Injury Scale (AIS)), GCS upon admission, length of hospital admission, length of intensive care unit (ICU) admission, number of days requiring ventilatory support, discharge status/mortality, rate of positive urine drug screen, and specific substances identified on positive urine drug screens.

Statistical calculations were performed using SAS 9.2 and R software, version R 3.6.3. The primary objectives of this study were to assess the association between trauma case load and severity of injuries and to assess incidence of substance abuse from the years 2009 to 2019. Descriptive statistics were used to summarize patient data. Categorical data was described using contingency tables including counts and percentages; continuous variables were summarized with descriptive statistical measures (i.e., mean (±SD) or median (range)). A Wilcoxon rank-sum test was used to assess the continuous variables between patients in 2009–2016 and 2017–2019 without normal distribution assumption, while a chi-square test was used to assess the association between two categorical variables. A *p* value of <0.05 implied statistical significance in this study.

## 3. Results

The study sample included 4,538 pediatric trauma patients aged from less than one month old to 18 years old at the time of presentation to a trauma center. 3,356 patients presented from 2009 to 2016, and 1,182 patients presented from 2017 to 2019. Mean age was 7.3 years from 2009 to 2016 and 6.7 years from 2017 to 2019. Males encompassed approximately 62–63% and females 37–38% of each group over the ten-year period. The most common trauma mechanisms stayed consistent over the ten-year span with blunt traumas accounting for 89-90% of presentations. This was followed by penetrating injuries (7-8%) and burn injuries (1-2%). GCS upon admission was statistically similar between both groups (14.5 versus 14.6, respectively).

Severity of injury was assessed using the AIS. Overall injury severity score (ISS) was 10.2 in patients with positive drug screens and 6.4 in those with negative drug screens (*p* < 0.001). The frequency of critical (AIS 5) head injuries increased from 3.7% in 2009–2016 to 7.2% in 2017–2019 (37/997 patients versus 23/318 patients, *p* = 0.007). Additionally, more severe head injuries (AIS 4 and AIS 5) were associated with positive urine drug screen results regardless of presentation year (*p* < 0.001). The frequency of serious (AIS 3) neck injuries increased from 12.2% to 27.1% in a similar pattern (14/115 patients versus 16/59 patients, *p* = 0.001). Moderate (AIS 2) lower extremity injuries decreased while minor (AIS 1) and serious (AIS 3) lower extremity injuries increased as time progressed (245/1,055 patients versus 109/372 patients, *p* < 0.001). Serious (AIS 3) lower extremity injuries were associated with positive urine drug screen results irrespective of presentation year (*p* = 0.001). The incidence of all other classifications of head, neck, and lower extremity trauma, as well as chest, abdomen, and upper extremity trauma, was statistically similar between the groups of patients analyzed. Injury data of statistical significance is summarized in Table 1.

Urine drug screens were obtained at a significantly higher rate during the latter half of the study, from 6.9% in 2009–2016 (231/3,356 cases) to 23.3% in 2017–2019 (275/1,182 cases) (OR = 4.10, *p* < 0.001). Of the urine drug screens that were collected, 2.1% were positive from 2009 to 2016 and 7.2% were positive from 2017 to 2019 (OR = 0.291, *p* < 0.001). The most identified substance during both intervals was oxycodone and other opioids, which displayed an increase in use from 1% in 2009–2016 to 4.9% in 2017–2019 (OR = 0.204, *p* < 0.001). The second most common substance identified was benzodiazepines, which displayed an increase in use from 0.8% to 1.8% (OR = 0.465, *p* < 0.001). Among other substances tested, there was no statistical difference in amphetamine use (0.1% increased to 0.4%, OR = 0.211, *p* = 0.051) or THC use (0.2% decreased to 0.1%, OR = 2.47, *p* = 0.638) as the years progressed. Drug screen data is summarized in Table 2.

The total number of hospital admission days for pediatric trauma cases increased from a mean of 2.4 days in 2009–2016 to 2.6 days in 2017–2019 (*p* < 0.001). Additionally, patients with positive urine drug screens had a longer duration of admission than those with negative urine drug screens (5.5 days versus 2.9 days, *p* < 0.001). Mean days requiring mechanical ventilation increased from 3.1 days to 6.3 days in a similar pattern (*p* < 0.001). The mean number of days spent in the ICU decreased in the later years, from 3.9 days to 3.7 days (*p* = 0.013). However, patients with positive urine drug screens had a longer duration of ICU admission than patients with negative tests (6.5 days versus 3.5 days, *p* < 0.001). Despite increased length of hospital stay and days requiring ventilation, morality rates were statistically similar between both groups but slightly higher in the later years (0.8% versus 1.3%, *p* = 0.17). Mortality rates were statistically similar between positive and negative urine drug screen groups, though sample size was small due to a high survival rate (approximately 99%). Patient outcome data is summarized in Table 3.

## 4. Discussion

The increasing use of illicit and prescription substances, paired with the increasing severity of pediatric trauma, is of significant concern in rural Appalachia. The substance use epidemic that the region is grappling with further exacerbates the disparities that exist in the area, some of which include lack of timely access to care due to limited availability of healthcare providers (Appalachia has 28% fewer specialty physicians than the national average) and lower socioeconomic statuses of the population (Appalachia has a median household income that is $10,000 less than the national average) [10]. Inhabitants of rural areas have lower life expectancies than their nonrural counterparts, and infant and child mortality has been shown to be higher in poor rural communities [11]. An analysis of over 16,000 trauma cases in Ohio revealed that nonurban areas experience pediatric trauma mortality rates that are four times higher than what should be expected [12]. Social issues endemic to rural Appalachia and many rural areas, as well as factors such as challenging geography and lack of medical presence in many communities, are contributing to these alarming trends. Conscious effort must be made to lessen these disparities in care.

Polysubstance toxicity with prescription sedatives such as benzodiazepines and opioids has frequently been observed in the adult rural population, particularly in younger white males with low education levels [13]. Based on the findings in our study, the effects of substance use in the adult population have had ramifications for the pediatric population. Our results indicate that the incidence of substance use discovered in cases of pediatric trauma continues to trend upward in rural Appalachia. Our findings revealed an overall positive drug screening rate of 7.2% from 2017 to 2019 in the region, which was similar to the positive screening rate of 8% in an Illinois study composed of over 12,000 patients [14]. Additionally, another previous study showed that trauma cases associated with a violent injury mechanism were more likely to screen positive for alcohol or drugs, most commonly THC or opioids [15]. Patients with violent injury mechanisms in our center's study suffered more severe head and neck injuries and were more likely to screen positive for opioids or benzodiazepines. Adult opioid overdose patients presenting to centers in the southern United States, including the Appalachian region, have required increased use of mechanical ventilation as time has progressed, which is similar to the increasing ventilation requirements we observed in our pediatric population as the years progressed [16]. The increased duration of hospital and ICU admission in pediatric patients testing positive for substances may pose many additional problems, ranging from financial burden on families to strain on resource-limited healthcare systems.

Efforts must be made to raise awareness of the substance use epidemic that is taking place in the pediatric population. Our health system's current protocol for pediatric trauma includes an automatic urine drug screening order for all patients who present. If the screen is positive, then patients over the age of twelve are evaluated with a validated substance use screening tool (CRAFFT) and by our Screening, Brief Intervention, and Referral to Treatment (SBIRT) team. This protocol, screening tool, and intervention have shown that substance abuse is indeed an issue in rural Appalachia. However, additional steps must be taken to ensure that the problem is adequately addressed beyond the initial trauma presentation. A previous study of 317 pediatric trauma cases who had urine drug screening revealed that only 39% of the positive tests were referred to services such as psychiatry or social work to address the substance misuse problem [8]. Early recognition of substance use in the adolescent population may be the most effective measure to prevent disease, as treatment of substance abuse in young individuals has proven to be difficult and has not been extensively studied [17]. Simple recognition of a potential drug-related problem is one area providers can improve in. There is often discordance with self-reported and charted substance use that may lead to decreased likelihood of treatment referral [18].

The rural areas impacted by the substance use epidemic must start to address the problem at the level of the pediatric population. Financial investment in education to increase awareness in school-aged children, providing easier access to comprehensive treatment facilities, and strengthening support systems that allow for stable social environments that prevent children from being placed back into futile situations are among some of the measures that may be needed. According to a recent literature review, programs such as Life Skills Training (LST) have shown to be effective in reducing adolescent use of substances such as alcohol and drugs in 70% of analyzed studies, whereas the more universally implemented Drug Abuse and Resistance Education (DARE) program has had no statistically significant impact on substance use in the pediatric population [19]. Therefore, further evaluation of these types of programs and possible redirection of funds towards the more effective ones may be necessary. Federal and state entities may also help by focusing on children in the 0–10-year-old age group to enable primary prevention of the issue rather than waiting until adolescence when the problem may have already developed. Addressing the increasing severity of the substance use epidemic and enacting change has the potential to occur at many levels, including with governments, communities, and at the level of the individual.

## 5. Conclusion

The increasing use of sedating, drive-depressing drugs such as opioids and benzodiazepines may be contributing to the increasing severity of head and neck trauma of the rural pediatric population. These results emphasize the importance of targeted interventions in populations that are predisposed to suffering from the endemic problems of the areas from which they are from. While the frequency at which drug screens were obtained in cases of pediatric trauma increased as the years progressed in our study, our results suggest that the current rate of just under 25% is still too low to effectively identify all patients in need of intervention. The automatic drug screening protocol currently in place by our system should help to rectify this problem. The ongoing substance and opioid use epidemics are not restricted to the adult demographic, so efforts must be made at multiple levels to preserve the quality and quantity of life in the rural pediatric population.

## Figures and Tables

**Table 1:** Injury severity in pediatric trauma cases by year.

AIS^∗^ classification		2009–2016 patients (%)	2017–2019 patients (%)	*p* value comparing injuries by year
Head injuries	AIS 5	37/997 (3.7)	23/318 (7.2)	0.007
Neck injuries	AIS 3	14/115 (12.2)	16/59 (27.1)	0.001
Lower extremity injuries	AIS 3 AIS 2 AIS 1	245/1055 (23.2) 378/1055 (35.8) 432/1055 (40.9)	109/372 (29.3) 83/372 (22.3) 179/372 (48.1)	<0.001 <0.001 <0.001

^∗^Abbreviated Injury Scale.

**Table 2:** Results of urine drug screens in pediatric trauma cases.

	2009–2016 patients (%)	2017–2019 patients (%)	Odds ratio	Chi-square *p* value
Amphetamines	3/3,356 (0.1)	5/1,182 (0.4)	0.211	0.051
Benzodiazepines	28/3,356 (0.8)	21/1,182 (1.8)	0.465	<0.001
Opioids/oxycodone	35/3,356 (1.0)	58/1,182 (4.9)	0.204	<0.001
Tetrahydrocannabinol (THC)	7/3,356 (0.2)	1/1,182 (0.1)	2.47	0.638
Drug screen positive for any substance	73/3,356 (2.1)	84/1,182 (7.2)	0.291	<0.001
Drug screen negative for all substances	158/3,356 (4.8)	190/1,182 (16.1)	0.258	<0.001
Drug screen not obtained in patient	3125/3,356 (93.1)	907/1,182 (76.7)	4.10	<0.001

**Table 3:** Patient information and outcome data for pediatric trauma cases.

	Total *n*	2009–2016 patients			2017–2019 patients			Wilcoxon rank test *p* value
		*n*	Mean	SD	*n*	Mean	SD	
Patient age (years)	4538	3356	7.3	4.5	1182	6.7	4.5	<0.001
GCS^∗^ on admission	4285	3104	14.5	2.3	1181	14.6	1.9	0.296
Total Hospital days	4538	3356	2.4	4.1	1182	2.6	4.7	<0.001
Injury severity score	4536	3354	5.615	5.65	1182	5.849	5.598	0.034
Total ICU^#^ days	898	704	3.9	5.4	194	3.7	5.6	0.013
Total ventilator days	220	169	3.1	5.1	51	6.3	10.3	<0.001

^∗^Glasgow Coma Scale; ^#^intensive care unit.

## Data Availability

Data is available upon request by contacting the corresponding author Pavithra Ellison, pavithra.ellison@hsc.wvu.edu.

## References

[1] About the Appalachian Region Appalachian Regional Commission. https://www.arc.gov/about-the-appalachian-region/.

[2] Ellison P., Cifarelli D., Pearce A. (2021). Incidence, prevalence, and outcomes of pediatric trauma in rural Appalachia (West Virginia) from 2017 to 2019. *Cureus*.

[3] Hswen Y., Zhang A., Freifeld C., Brownstein J. S. (2020). Evaluation of volume of news reporting and opioid-related deaths in the United States: comparative analysis study of geographic and socioeconomic differences. *Journal of Medical Internet Research*.

[4] Hale J. V., Feyh A. S., Weaver A., Murray J., Denning D. A., Amiri F. (2020). The effect of substance abuse programs on positive drug screening tests in trauma patients. *The American Surgeon*.

[5] Winstanley E. L., Stover A. N. (2019). The impact of the opioid epidemic on children and adolescents. *Clinical Therapeutics*.

[6] National Trauma Data Bank 2016 Pediatric annual report. https://www.facs.org/~/media/files/quality%20programs/trauma/ntdb/ntdb%20pediatric%20annual%20report%202016.ashx.

[7] Maxwell B. G., Lin S., Greene N. H., Jafri M. A. (2021). Kids grow up so fast: national patterns of positive drug/alcohol screens among pediatric trauma patients. *Pediatric Research*.

[8] Robinson T., Tarzi C., Zhou X. G., Bailey K. (2020). Screening for alcohol and substance use in pediatric trauma patients: a retrospective review. *Journal of Pediatric Surgery*.

[9] Merino R., Bowden N., Katamneni S., Coustasse A. (2019). The opioid epidemic in West Virginia. *Health Care Management*.

[10] Marshall J., Thomas L., Lane N., Holmes G. M., Arcury T., Randolph R. Health disparities in Appalachia. https://www.arc.gov/wp-content/uploads/2020/06/Health_Disparities_in_Appalachia_August_2017.pdf.

[11] Singh G. K., Daus G. P., Allender M. (2017). Social determinants of health in the United States: addressing major health inequality trends for the nation, 1935-2016. *International Journal of MCH and AIDS*.

[12] Ertl A. M., Beyer K. M. M., Tarima S., Zhou Y., Groner J. I., Cassidy L. D. (2017). The spatial epidemiology of pediatric trauma: a statewide assessment. *Journal of Trauma and Acute Care Surgery*.

[13] Schalkoff C. A., Lancaster K. E., Gaynes B. N. (2020). The opioid and related drug epidemics in rural Appalachia: a systematic review of populations affected, risk factors, and infectious diseases. *Subst Abuse*.

[14] Nicolson N. G., Lank P. M., Crandall M. L. (2014). Emergency department alcohol and drug screening for Illinois pediatric trauma patients, 1999 to 2009. *American Journal of Surgery*.

[15] Noffsinger D. L., Wurster L. A., Cooley J. (2019). Alcohol and drug screening of adolescent trauma alert patients at a level 1 pediatric trauma center. *The American Journal of Emergency Medicine*.

[16] Oladunjoye A. O., Oladunjoye O. O., Olubiyi O., Yee M. R., Espiridion E. D. (2020). Predictors and outcomes of invasive mechanical ventilation in opioid overdose hospitalization in the United States. *Cureus*.

[17] Stockings E., Hall W. D., Lynskey M. (2016). Prevention, early intervention, harm reduction, and treatment of substance use in young people. *Lancet Psychiatry*.

[18] Masonbrink A. R., Hunt J. A., Bhandal A. (2021). Self-reported and documented substance use among adolescents in the pediatric hospital. *Pediatrics*.

[19] Tremblay M., Baydala L., Khan M. (2020). Primary substance use prevention programs for children and youth: a systematic review. *Pediatrics*.

